# Growing up with HIV

**DOI:** 10.1002/jia2.25606

**Published:** 2020-08-10

**Authors:** Carey Pike, Lwandile Sigaqa

**Affiliations:** ^1^ Desmond Tutu HIV Foundation Cape Town South Africa; ^2^ Youth mentor Adolescent Health Programmes Desmond Tutu HIV Foundation Cape Town South Africa

**Keywords:** youth, HIV, youth engagement, Adolescent girls and young women, Adolescents, Community, Key and vulnerable populations, International Youth Day

Our perspective and experience of HIV is fundamentally shaped by our age. HIV emerged in 1981 and we, the millennials and all subsequent generations, were brought up in a world where technology, globalization and HIV already existed. We know these were game‐changers and we fully experience their impact – but we cannot imagine a world without them. Youth, the 15‐ to 24‐year‐olds of today, have always faced HIV with confirmed risk factors and efficacious treatment options. Although access to HIV treatment and prevention services remains highly variable, the technology, medicine and evidence to support them are available.[1] Given this context, what then are our priorities as youth living with and without HIV?

The United Nation’s 2020 International Youth Day is set to highlight the impact of youth engagement, an endeavour that has been well‐supported within the HIV field.[2] There are continued initiatives to make youth engagement in HIV research more meaningful, as well as to enhance youth representation at major sector events.[3, 4, 5] These efforts are not unique to HIV and across most global issues youth are increasingly being heard, prioritized and finding power and inspiration through their involvement.[6, 7, 8]

What are our HIV priorities and why would they differ from other vulnerable populations or age groups? Despite tremendous efforts towards achieving an HIV‐free generation, HIV remains a concern among the youth as a leading cause of global mortality alongside sustained high infection rates, particularly among African youth, young women and young key populations.[9, 10] In 2019, 24% of all HIV infections in sub‐Saharan Africa occurred among adolescent girls and young women.[11]

Youth living with HIV face a lifetime of treatment management and disclosure that will see them navigate all of life’s major milestones, including finding work, starting a family and old age.[12] The availability of evidence‐based HIV treatment and prevention options shifts our priorities away from the disease and its remedies, to finding ways to better facilitate the services that surround them. As youth, we expect the world to be non‐judgemental, having been convinced we can #beyourself, let #youdoyou and that #loveislove. So we are surprised when healthcare professionals are judgemental, unfriendly, or turn us away. We expect things in our world to be accessible at times that are convenient for us, as shopping centres are, so we are surprised when healthcare services are not available after school, after hours, or on weekends. The youth want to see convenient, non‐judgemental services prioritized and delivered. Yet despite support from research and the available WHO guidelines, widespread youth‐friendly services remain elusive for many.[13, 14]

Our familiarity with HIV acts to bring it in line with a number of other youth‐associated health concerns, such as diabetes and becoming pregnant, that may feel as, or sometimes more, concerning.[12, 15] As a generation that is used to owning one mobile device that caters to a full spectrum of needs and an online portal that is simultaneously our library, shopping mall and social club, having our health needs separated into different visits, clinics and professionals seems odd and difficult to manage. Integrated, one‐stop‐shops, that preferentially have an online option, are what we want but also expect.[1] The movement of the World Health Organization towards Universal Healthcare delivery, the numerous examples of where this has already been achieved, and the enhanced global access to technology (particularly among youth and especially within a virtualized COVID 19 world) make integrated care a high priority.[1, 16]

Our familiarity with HIV and the range of treatment and prevention options available means that HIV is seldom a young person’s stand‐alone priority. Our generation is heavily invested in many of the world’s current major social battles, including Black Lives Matter, gender‐based violence and the Me Too movement, LGBQ + rights and Climate Change. Many youth activists are channelling their energy into these movements. However, this does not mean HIV is being left behind. HIV, as a virus that picks on the marginalized and oppressed, thrives off the social problems that these movements seek to change and so is likely to benefit if gains are made against these social ills – or equally suffer from any losses. What it does mean is that increasing support for these youth‐invested movements from within the HIV response could help attract youth into the HIV arena and showcase its continued and very real relevance. As emphasized in the July 2020 UNAIDS report, we cannot afford to lose momentum in the fight to end HIV. However, we do need to consider how to draw the next generation into leading that fight.[11]

Our familiarity with HIV means that as youth we struggle with being told that living with HIV means we are different. Youth, who have integrated HIV into their world, want to feel integrated too. This necessitates the accelerated pursuit of seroneutral, integrated programmes and services, where an HIV positive status does not make you an exception. Youth living with HIV nevertheless do need support to maintain adherence to treatment as well as navigate the transition to adult healthcare. In addition, despite the fact that our online generation does not shy away from disclosing personal information, a lifetime of HIV status disclosure and explanations can lead to disclosure fatigue.[17] The enhanced availability of consistent practical and mental health support via adherence clubs and counselling services, preferentially ones that are community‐based and peer‐led, as well as continued pursuit of acceptable and effective mobile applications should be prioritized.[18, 19, 20]

We, the youth, are familiar with HIV and its services. Now, we want to help champion the move towards making those services youth‐friendly, seroneutral, integrated with our other numerous health and social concerns, and, where possible, virtual.

## COMPETING INTERESTS

The authors have no conflicts of interest to declare.

## AUTHORS’ CONTRIBUTIONS

CP and LS jointly conceptualized the viewpoint. CP wrote the first draft. LS reviewed and refined the piece. Both authors read and approved the final manuscript.
